# Racial differences in tolerability of topical retinoids: A 15-year single-center retrospective cohort study

**DOI:** 10.1016/j.jdin.2024.04.012

**Published:** 2024-05-09

**Authors:** Yu-Feng Chang, Li-Chi Chen, Dae Hyun Kim, Sarah Hahn Hsu, Hye Jin Chung

**Affiliations:** a Department of Dermatology, Beth Israel Deaconess Medical Center, Boston, Massachusetts; b Harvard Medical School, Boston, Massachusetts; c Harvard T.H. Chan School of Public Health, Boston, Massachusetts; d Hinda and Arthur Marcus Institute for Aging Research, Hebrew Senior Life, Boston, Massachusetts; e Department of Dermatology, Johns Hopkins University School of Medicine, Maryland

**Keywords:** acne vulgaris, cutaneous irritation, intolerability, race, tolerability, topical retinoids

*To the Editor:* Skin barrier function and tolerability may vary among different races because of structural and functional skin differences.^1^, ^2^, ^3^ Topical retinoids often cause irritation, leading to early termination of treatment. Despite the perception of greater irritability to topical agents in Asian skin because of more fragile skin barriers than those of other races,^4^ to our knowledge, no studies have explored the racial differences in the tolerability of topical retinoids. Our study aimed to evaluate the association between various racial groups and the tolerability of topical retinoids.

This retrospective cohort study used electronic medical records at Beth Israel Deaconess Medical Center from 2006 to 2020 and identified 1456 patients aged ≥18 years prescribed topical retinoids for acne who self-identified as White, Black, or Asian. We excluded 312 patients without follow-up visits within 2 years of first prescription. A total of 3 racial cohorts of 753 patients (*n* = 251 per race group) matched on age, sex, and topical retinoid prescription. We also recorded their insurance status. The primary outcome was intolerability to topical retinoids based on clinical documentation of local irritations such as burning, pruritus, erythema, dryness, and peeling. Multivariable regression models were used to examine the association between self-reported race and intolerability by adjusting for age, sex, insurance status, and initial prescription. A sensitivity analysis was performed with multiple imputations (10 imputations) to impute missing outcome data for patients with no follow-up visit on intolerability. A 2-sided *P* value of <.05 was considered statistically significant. All analyses were performed using Stata IC 16.1 (StataCorp LLC).

Our study population included 753 patients with acne (mean age ± SD, 29.5 ± 9.0 years; 639 [84.9%] females) whose tolerability data were available (Table I). In a multivariate analysis, the odds of intolerability were lower in Black (multivariable-adjusted odds ratio [95% CI], 0.25 [0.11-0.57]; *P* = .001) than in Asian population (Table II). There was a statistically nonsignificant trend toward lower intolerability in White than in Asian patients (*P* = .109). Multiple imputation analyses of missing outcomes revealed similar results for Black (multivariable-adjusted odds ratio [95% CI], 0.28 [0.12-0.61]; *P* = .002) and Asian populations.

**Table I:** Patient characteristics

Variable	Total (*N =* 753)	White (*n =* 251)	Black (*n =* 251)	Asian (*n =* 251)	*P* value
Age (y), mean ± SD	29.5 ± 9.0	29.7 ± 9.7	29.5 ± 8.2	29.3 ± 9.0	.835
Age group (y), *n* (%)					1.000
18-25	300 (39.8)	100 (39.8)	100 (39.8)	100 (39.8)	
26-35	318 (42.2)	106 (42.2)	106 (42.2)	106 (42.2)	
36-45	96 (12.8)	32 (12.8)	32 (12.8)	32 (12.8)	
≥46	39 (5.2)	13 (5.2)	13 (5.2)	13 (5.2)	
Sex, *n* (%)					1.000
Female	639 (84.9)	213 (84.9)	213 (84.9)	213 (84.9)	
Male	114 (15.1)	38 (15.1)	38 (15.1)	38 (15.1)	
Insurance, *n* (%)					.092
Commercial	491 (65.2)	207 (82.4)	119 (47.4)	165 (65.7)	
Public	233 (30.9)	34 (13.6)	123 (49.0)	76 (30.3)	
Self-insured	29 (3.9)	10 (4.0)	9 (3.6)	10 (4.0)	
Initial regimen, *n* (%)					1.000
Tretinoin 0.025% cream	603 (80.1)	201 (80.1)	201 (80.1)	201 (80.1)	
Tretinoin 0.05% cream	105 (13.9)	35 (13.9)	35 (13.9)	35 (13.9)	
Tretinoin 0.1% cream	24 (3.2)	8 (3.2)	8 (3.2)	8 (3.2)	
Other retinoids∗	21 (2.8)	7 (2.8)	7 (2.8)	7 (2.8)	

The χ^2^ test was used for categorical variables. Analysis of variance was used to measure continuous variables among different racial groups.

∗ Other retinoids include adapalene 0.1% gel, adapalene 0.3% gel, tazarotene 0.05% cream, tazarotene 0.1% cream, tretinoin microspheres 0.01% gel, and tretinoin microspheres 0.04% gel.

**Table II:** Logistic regressions for associations between patients’ self-reported race and intolerability of topical retinoid treatment over 24 months (*N =* 753)

Variable	Intolerability (%)	Univariate model		Multivariable model	
		OR (95% CI)	*P* value	OR (95% CI)	*P* value
Race					
Asian	31/251 (12.4)	1.00 (Reference)		1.00 (Reference)	
White	22/251 (8.8)	0.68 (0.38-1.21)	.193	0.62 (0.34-1.11)	.109
Black	8/251 (3.2)	0.23 (0.11-0.52)	**<.001**	0.25 (0.11-0.57)	**.001**
Age group (y)					
18-25	32/300 (10.7)	1.00 (Reference)		1.00 (Reference)	
26-35	20/318 (6.3)	0.56 (0.31-1.01)	.053	0.51 (0.28-0.93)	**.029**
36-45	6/96 (6.3)	0.56 (0.23-1.38)	.206	0.53 (0.21-1.34)	.178
≥46	3/39 (7.7)	0.70 (0.20-2.40)	.568	0.93 (0.26-3.35)	.909
Sex					
Female	57/639 (8.9)	1.00 (Reference)		1.00 (Reference)	
Male	4/114 (3.5)	0.37 (0.13-1.04)	.060	0.34 (0.12-0.97)	**.043**
Insurance type					
Commercial	49/491 (10.0)	1.00 (Reference)		1.00 (Reference)	
Public	10/233 (4.3)	0.40 (0.20-0.81)	**.011**	0.51 (0.25-1.06)	.072
Self-insured	2/29 (6.9)	0.67 (0.15-2.90)	.590	0.55 (0.12-2.45)	.431
Initial regimen					
Tretinoin 0.025%	48/603 (8.0)	1.00 (Reference)		1.00 (Reference)	
Tretinoin 0.05%	7/105 (6.7)	0.83 (0.36-1.88)	.648	0.85 (0.36-1.96)	.695
Tretinoin 0.1%	5/24 (20.8)	3.04 (1.09-8.51)	**.034**	2.97 (1.01-8.74)	**.048**
Other retinoids	1/21 (4.8)	0.58 (0.08-4.40)	.597	0.65 (0.08-5.06)	.677

Bold values denotes statistical significance at the *P* < .05 level.

*OR*, Odds ratio.

Our study demonstrated that Asian patients may be less likely to tolerate topical retinoids compared with Black patients, with a similar trend observed when compared with White, albeit not statistically significant. This finding aligns with the previous studies indicating that Asian population has greater reactivity to irritants.^2^^,^^3^ Additionally, males demonstrated better tolerance to topical retinoids than females (*P* = .043), consistent with previously reported gender-related differences in skin physiology.^5^ This study suggests that race and gender differences should be incorporated in the management of acne, alongside strategies to minimize irritation and enhance adherence to topical retinoids. Limitations of our study include a high rate of loss to follow-up (21.4%), a single-center design, and the exclusive focus on the tolerability in patients with acne, limiting applicability of our findings to broader dermatologic issues such as postinflammatory hyperpigmentation. The clinical documentation of intolerability may be influenced by patients’ likelihood to show up and physicians’ likelihood to assess them. Hispanic patients were omitted because of their small sample size. Further prospective trials are required to optimize retinoid regimens across diverse patient populations.

## Conflicts of interest

None disclosed.
